# Effects of fermentation time on the functional and pasting properties of defatted *Moringa oleifera* seed flour

**DOI:** 10.1002/fsn3.262

**Published:** 2015-07-30

**Authors:** Omobolanle O. Oloyede, Samaila James, Ocheme B. Ocheme, Chiemela E. Chinma, V. Eleojo Akpa

**Affiliations:** ^1^ Department of Food Science and TechnologyFederal University of TechnologyPMB 65MinnaNiger StateNigeria

**Keywords:** Fermentation, functional, Moringa seed, pasting, solvent extractionsolvent extraction

## Abstract

Effects of fermentation time on the functional and pasting properties of defatted *Moringa oleifera* seed flour was examined. Moringa seeds were fermented naturally at 0, 12, 24, 48 and 72 h; oven dried at 60°C for 12 h; milled into five different flour samples for each fermentation time and defatted. The functional and pasting properties of the samples were determined. The result shows significant increase in the water absorption capacity, oil absorption capacity, foaming capacity and emulsifying capacity with increase in fermentation time. However, there was a significant decrease in bulk density (0.53–0.32 g/cm^3^) and dispersibility (36.00–20.50%) with an increase in fermentation time. There were significant increase in peak viscosity, trough, breakdown, final viscosity, and set back with increasing fermentation time. The swelling power and solubility of fermented Moringa seed flour was significantly affected.

## Introduction

The insufficiency of animal protein and costliness of few available plant sources have prompted intense research in the area of local and underutilized vegetable plant proteins and legume seeds. The efforts would significantly improve nutrition and house hold food security (National Research Council, 2006).

*Moringa oleifera* is an important underutilized traditional vegetable tree widely cultivated in India and many countries in tropical Africa (Morton 1991; Anjorin et al. 2010; Ogunsina et al.*,*). It is commonly known as drum stick tree or horseradish tree in English and locally known as *Zogale* among the Hausa‐speaking people of Nigeria (Anjorin et al. 2010). *Moringa* possesses many valuable properties, which is of great scientific interest. This includes the high‐protein content (36.18%) in the seed which is not only abundant in good essential amino acids, but can be used to supplement cereal and tubers (Foidl et al. 2001). It is also rich in fat and oil, provitamins, and minerals as compared to most fruits. (Makkah and Becker 1997; Ogunsina et al., ).

*Moringa* is regarded as a versatile plant due to its multiple uses (Anwar and Rashid 2007). The incorporation of Moringa seed flour into maize flour for making cookies has been reported (Aluko et al. 2011). Different parts of the plant are edible and used as food. They include the leaves which are cooked and eaten like spinach, salad, or to make soup. The young green pods are boiled and eaten like green beans and the dry seeds are roasted and eaten like peas. Moringa seed is ground in to flour and used domestically in soup seasoning and industrially used as a flocculating agent for water purification(Gassenchmidt et al., 2005). Oil from the seed is used for cooking and as a solidifying agent in margarine production and other foodstuff that contain solid and semisolid fats thereby eliminating the hydrogenation process (FDA 2001).

Fermentation is an effective processing method used to improve nutritional quality of plant food as well as eliminating antinutritional factors (Steinkraus 1995). Fermentation improves digestibility, extends shelf life, and enhances the flavor and taste of raw seed (Achi and Okereke 1999).

Solvent extraction is a unit operation that involves separation of specific component of food, such as the removal of a desired component (the solvent) which is able to dissolve the solute (Clarke 1990). The solvent n‐hexane is commonly used due to its extraction efficiency and ease of availability. It is preferred due to its low boiling point, low greasy residual, low corrosiveness, and high stability (Niosh 2007).

Processing methods tend to affect the characteristic of protein, carbohydrate, lipid, and their behavior in food systems as well as sensory properties of foods. The application of food flour in food systems depends largely on the knowledge of their functional properties. This is because of their influence on textural properties, sensory attributes, and consumer acceptability on their finished products (Adebowale et al. 2005; James and Nwabueze 2014). It is therefore necessary to study the effect of fermentation on the functional and pasting properties of defatted Moringa seed flour. The expected results would suggest the food applicability of the treated flour thereby expanding its usage.

## Materials and Methods

### Source of materials

Moringa seeds used for the study were purchased from Kure Ultra‐Modern Market, Minna, Niger State, Nigeria.

### Raw material preparation

#### Preparation of fermented *Moringa* seed flour

The method described by Oluwafemi and Ikeowa (2005) was adopted for the preparation of fermented *Moringa* flour with slight modification which was drying the fermented seeds before milling. About 100 grams of fresh *Moringa* seeds was put into four different 1000 mL beakers, and 500 mL of distilled water was added to each beaker. Moringa seeds were fermented for 12, 24, 48, and 72 h, respectively, at a room temperature 29 ± 02°C. At the end of the fermentation period, the seeds were washed with tap water, drained, and dried in a Gallenkamp oven at 60°C for 12 h. The dried seeds were then milled to flour and passed through 100 μm mesh size. The resultant flour from each fermentation time was coded and stored in airtight containers at 4°C for further processing. The untreated *Moringa* flour was used as control.

#### Solvent extraction

The method described by Nwabueze and Iwe (2010) was adopted. Each flour sample was placed in a food grade hexane at 1:3 (flour: n‐hexane) ratio for 3 h at room temperature and centrifuged at 3000 rpm for 10 min. The flour was separated from the supernatant and spread under the fan in order to remove the residual solvent (hexane) in the sample. The defatted flour was dried in an air convection oven (Gallenkamp, England) at 60°C for 12 h to further reduce the moisture level. The resultant defatted flour were milled using a hammer mill in order to break flour lumps and stored in an airtight container in the refrigerator at 4°C for further analysis.

### Methods

#### Functional properties

The bulk density, oil, and water absorption capacity were determined by the method described by Adebowale et al. (2005). The dispersibility of the flour samples was measured by the method described by Kulkarin et al. (1991). The method described by Yatsumatsu et al. (1992) was used to determine the emulsion capacity. Foam capacity was determined by the method of Adebowale et al. (2005).

#### Determination of pasting properties of *Moringa* seed flour

Pasting parameters were determined using rapid visco analyzer (Newport Scientific Pty Ltd., Warrie‐Wood, NSW, Australia). About 2.5 g of the flour sample was weighed into a dried empty canister; then 25 mL of distilled water was dispensed into the canister containing the sample. The suspension was thoroughly mixed and the canister was fitted into the rapid visco analyzer. Each suspension was kept at 50°C for 1 min and then heated up to 95°C at 12.2°C/min and held for 2.5 min at 95°C. It was then cooled to 50°C at 11.8°C/min and kept for 2 min at 50°C.

#### Determination of swelling power and solubility

The method of Sathe and Salunkle () was used to determine the swelling power and solubility of the flour samples with slight modification in temperature. One gram of the flour sample was weighed into a previously tarred 50 mL centrifuge tube and 40 mL of 1% starch suspension (w/v) was added. The slurry was heated in a water bath at 60, 80, and 100°C, respectively for 15 min. During heating, the slurry was stirred gently to prevent clumping of the starch. After 15 min, the tubes containing the slurry were centrifuged at 3000 rpm for 10 min. The supernatant was decanted and the swollen granules weighed. A 10 mL sample was taken from the supernatant, placed in a crucible, and dried in an air convention oven at 120°C for 4 h to constant weight. The average value of the triplicate determinations was recorded.

### Statistical analysis

Data were analysed using analysis of variance (Steel and Torrie 1980). The difference between mean values was determined by the least significant different test. Significance was accepted at the 5% probability level.

## Results and Discussion

### Functional properties of fermented seed *Moringa* flours

The functional properties of defatted *Moringa* flour samples are shown in Table 1. The result shows that water absorption capacity of the flour at different fermentation times was significantly (*P* < 0.05) higher than the control. This implies that there was an increase in water absorption capacity with increasing fermentation time. Gomez and Aguilera (1983) explained that low water absorption capacity value for raw sample is an indication of intact starch granules in the raw flour. The result of this study compares favorably with 1.16 g/mL and 1.31 g/mL for full fat and defatted Moringa seed flour, respectively (Ogunsina et al. 2014). Furthermore, the result shows similar trend reported by Alfaro et al. (2004); Filli et al. (2010) and James and Nwabueze (2014) for soya bean flour, extruded millet‐soybean flour, and extruded African breadfruit flour mix, respectively. The increase in water absorption capacity of Moringa seed flour could be due to the modification of macromolecules during fermentation. The modification exposes the hydrophilic domains of macromolecules which have high affinity for water. The significantly high value (2.31 g/mL) at the 48 h fermentation time represents the peak of bio modification. The high value recorded in this study implies the suitability of the flour and its isolates for incorporation into aqueous food formulation especially those involving dough handling.

**Table 1:** Effect of fermentation time on the functional properties of Moringa seed flour

Functional properties	A	B	C	D	E
Water‐holding capacity (g/mL)	0.86^c^ ± 0.05	1.54^b^ ± 0.01	1.59^b^ ± 0.12	2.31^a^ ± 0.33	1.81^b^ ± 0.12
Oil‐binding capacity (g/mL)	1.02^c^ ± 0.02	0.87^d^ ± 0.01	1.69^b^ ± 0.02	1.91^a^ ± 0.02	1.68^b^ ± 0.014
Emulsifying capacity (%)	50.71^e^ ± 0.01	60.85^d^ ± 0.21	65.16^c^ ± 0.31	65.96^b^ ± 0.06	68.75^a^ ± 0.01
Foaming capacity (%)	9.90^c^ ± 0.14	16.31^a^ ± 0.51	9.76^c^ ± 0.06	13.87^b^ ± 0.19	9.84^c^ ± 0.05
Dispersibility (%)	36.00^a^ ± 1.414	21.50^d^ ± 0.71	24.00^c^ ± 0.00	29.00^b^ ± 0.00	20.50^d^ ± 0.71
Bulk density (g/cm^3^)	0.60^a^ ± 0.01	0.32^b^ ± 0.01	0.37^b^ ± 0.04	0.39^b^ ± 0.02	0.54^a^ ± 0.08

Values are means and standard deviation of two determinations.

Value followed by the same superscript letters in a column are not significantly (*P* > 0.05) different.

Keys: A = Control (unfermented Moringa flour); B = 12 h fermented Moringa flour; C = 24 h fermented Moringa flour; D = 48 h fermented Moringa flour; E = 72 h fermented Moringa flour.

The oil absorption capacity of the flour samples ranged from 0.87 to 1.91 g/mL. There was a significant (*P* < 0.05) increase in the oil absorption capacity of the flour samples with increase in fermentation time. Sample D had the highest value (1.91 g/mL). The oil‐binding capacity reported in this study compares favorably with 1.30 and 2.08 g/mL for full fat and defatted Moringa seed flour, respectively (Ogunsina et al. 2014). The result is also in line with Periago et al. (1998) who reported that pea flour showed an increase in oil absorption capacity with fermentation time. According to Fagbemi (1999), good oil absorption capacity of flour samples suggest that they may be useful in food preparations that involve oil mixing as in bakery products, where oil is an important ingredient. Therefore, Moringa seed flour may be used to replace some legumes and oil seeds as thickeners in some liquid and semiliquid food reparations. It can also act as a flavor retainer, which implies that it can be incorporated in food for improved taste.

The emulsion capacity of fermented Moringa flour ranged from 50.71% to 68.75%. The result in this study agrees with Ogunsina et al. (2014) for raw Moringa seed flour however, low compared with 97.2% for defatted sample. Sample E was significantly (*P* < 0.05) high in the emulsion capacity while the control was significantly (*P* < 0.05) low. The emulsion capacity increased with increase in fermentation time. Emulsion capacity indicates the maximum amount of oil that can be emulsified by protein dispersion, whereas emulsion stability indicates the ability of an emulsion with a certain composition to remain unchanged (Enujiugha et al. 2003). Emulsion capacity is an important consideration in the production of pastries and frozen desserts. Therefore, Moringa flour may thus be useful in such food formulations. The increasing trend in emulsion capacity is an indication that the flour is suitable in making cake batter, mayonnaise, and salad dressing among others.

The foaming capacity of fermented Moringa flour significantly (*P* < 0.05) increased during the 12 h fermentation time. However, during the 24 and 72 h fermentation periods, the value significantly (*P* < 0.05) reduced. Foaming capacity has been reported to improve the texture, consistency, and appearance of foods (Akubor and Chukwu 1999). Foam formation and stability are dependent on pH, viscosity, surface tension, and processing methods. The foaming properties recorded in this study is higher than those recorded for pumpkin by Oshodi and Fagbemi (1992) and germinated tiger nut varieties reported by Chinma et al. (2009). The trend in the foam capacity in this study agrees with James and Nwabueze (2014) for extruded African breadfruit flour mix. The foam capacity at 12 h fermentation time (16.31%) compares favorably with 20.60% for raw Moringa seed flour, however, the value was found to be high compared to 9.90% for the same raw Moringa seed flour reported in this study. The difference could be attributed to species variation and climatic difference among others. High foaming capacity by samples B and D implies that the flour samples could be used for leavening food products such as baked food, cakes, and biscuits.

The bulk density ranged from 0.32 to 0.59 g/cm^3^. There was significant (*P* < 0.05) decrease in the bulk density of the flour samples with respect to fermentation time. However, at 72 h fermentation period (sample E), the value significantly increased. The bulk density of a flour sample influences the amount and the strength of packaging material; texture, and mouth feel (Wilhelm et al. 2004). Therefore, the low value of bulk density obtained from this study makes the samples desirable for packaging.

### Pasting properties of fermented *Moringa* seed flours

Pasting properties of flours are parameters used in determining the suitability of its application as functional ingredient in food and other industrial products. The effect of fermentation on the pasting properties of *Moringa* flour is presented in Table 2. Peak viscosity value ranged from 15.00 to 34.00 RVU. Peak viscosity is the ability of starch to swell freely before their physical break down. Peak viscosity indicates the water‐binding capacity of starch. It generally depends on solubility and water‐holding capacity as well as the structure of components in a food system (Leszek 2011). Sample C had the highest value while sample D had the least value. The value increased with increasing fermentation time at 12 and 24 h fermentation periods however, significantly (*P* < 0.05) reduced during the 48 h fermentation time. Studies have shown that flour with lower peak viscosity have a lower thickening power than flour with high peak viscosity which is attributed to many factors. Egouletey and Aworh (1991) reported that protein and fat interaction in the blend of African yam, beans, and cassava starch lowers the peak viscosity. Furthermore, Belitz and Grosch (1999) and James and Nwabueze (2014) reported that extrusion cooking lowers peak viscosity of extrudates. Therefore, to increase the pasting characteristics of Moringa flour, there is need for the flour to be blended with other high pasting flours.

**Table 2:** Pasting properties of fermented Moringa seed flour

Pasting properties (RVU)	A	B	C	D	E
Trough	17.00^b^ ± 1.41	17.00^b^ ± 1.41	21.50^a^ ± 0.71	11.00^c^ ± 0.00	18.50^b^ ± 0.71
Breakdown	7.00^b^ ± 1.41	13.50^a^ ± 0.71	12.50^a^ ± 0.71	4.00^c^ ± 0.00	14.50^a^ ± 0.71
Final viscosity	27.50^b^ ± 2.12	30.00^b^ ± 1.41	34.50^a^ ± 0.71	16.00^c^ ± 0.00	36.00^a^ ± 0.00
Setback	10.50^c^ ± 0.71	13.00^b^ ± 0.00	13.00^b^ ± 0.00	5.00^d^ ± 0.00	17.50^a^ ± 0.00
Peak time	4.93^a^ ± 0.94	4.37^a^ ± 0.05	4.90^a^ ± 0.05	4.57^a^ ± 0.24	4.23^a^ ± 0.05
Peak	24.00^b^ ± 2.83	30.50^a^ ± 0.71	34.00^a^ ± 1.41	15.00^c^ ± 0.00	33.00^a^ ± 1.41

Values are means and standard deviation of two determinations.

Value followed by the same superscript letters in a column are not significantly (*p* > 0.05) different.

Keys: A = Control (unfermented Moringa flour); B = 12 h fermented Moringa flour; C = 24 h fermented Moringa flour; D = 48 h fermented Moringa flour; E = 72 h fermented Moringa flour.

The final viscosity of the flour samples ranged from 16.00 to 36.00 RVU. Sample E had the highest value while sample D the lowest value. Shimel et al. (2006) reported that final viscosity of flour sample is the ability of starch to form paste and gel during cooking and cooling. The final viscosity of starch paste is related to the amylose content. This implies that the flour with a higher amylose content gives a higher viscosity and flour with a lower amylose content gives a lower viscosity. The final viscosity significantly (p < 0.05) increased at the 24 h fermentation period but, significantly reduced at the 48 h and latter increased at the 72 h fermentation period. The increase could be attributed to the breakdown of complex carbohydrates to lower sugars during fermentation.

Setback is related to the amylose content and reflects the retrogradation of starch. The setback values of the flour samples ranged from 5.00 to 17.50 RVU. Sample E (72 h fermentation time) was significantly (*P* < 0.05) high while, sample D (48 h fermentation time) was significantly (*P* < 0.05) low. The higher the setback value, the lower the retrogradation during cooling of the product made from the flour (James and Nwabueze 2014). This implies that Moringa seed flour fermented for 3 days would have high gel stability compared with others. Trough viscosity is the maximum viscosity value at the constant temperature phase of the RVU profile and measures the ability to withstand breakdown during cooling (Chinma et al. 2009). The trough value ranged from 11.00 to 21.50 RVU. Sample C was found to be significantly (*P* < 0.05) high in trough viscosity. Fermentation time significantly (*P* < 0.05) increased the trough at 24 h while at 48 h the fermentation time significantly (*P* < 0.05) reduced.

The breakdown value ranged from 4.00 to 14.50 RVU. The higher the break down viscosity, the lower the ability of the sample to withstand heating and shear stress during cooking (Adebowale et al. 2005). Fermentation time significantly (*P* < 0.05) increased the breakdown values of the flour samples except in sample D. The result suggests that increased breakdown value with increase in fermentation time would enable easy cooking but, susceptible to stress when processed into the solid form. Breakdown viscosity value is a measure of ease with which the swollen granules can be disintegrated and hence an indicator of the stability of the flour product (Kaur and Singh 2005).

The pasting time of the flour samples ranged from 4.23 to 4.93 min. Fermentation time did not significantly (*P* > 0.05) affect the pasting time of the flour samples. Pasting time is a measure of the minimum time and temperature required to cook flour (Chinma et al. 2009). The pasting time was statistically similar for all the samples. This implies that the fermentation time has no influence on the pasting time of raw and fermented Moringa seed flour.

### Swelling power of defatted Moringa seed flour at different temperatures

The swelling power of defatted Moringa seed flour at different temperatures is shown in Table 3. The value increased with an increase in fermentation time. Sample B (12 h) was significantly (*P* < 0.05) high while sample D was significantly (*P* < 0.05) low. Increased swelling power in fermented samples could be due to modification of starch granules during fermentation, resulting in higher water uptake by the granules. Claver et al. () reported that temperature increase and vigorous starch vibration break intermolecular bonds, thereby allowing hydrogen bonding sites to accommodate more water molecules. At 80°C, the swelling power of the flour samples has no significant (*P* > 0.05) difference. This implies that fermentation time had no influence on swelling power at different temperatures. At 100°C, the swelling power decreased with increase in fermentation time with the exception of sample B which is significantly higher.

**Table 3:** Effect of fermentation time on the swelling power of Moringa seed flour

Temperature (°C)	Swelling power				
	A	B	C	D	E
60	0.88^e^ ± 0.06	80.78ª ± 0.08	3.62^c^ ± 0.03	2.12d ± 0.02	3.95^b^ ± 0.08
80	6.33ª ± 7.00	10.18ª ± 0.10	5.61ª ± 0.00	6.34^a^ ± 0.15	6.10ª ± 0.01
100	11.13^b^ ± 1.59	17.25ª ± 0.00	6.69^c^ ± 0.04	7.07^c^ ± 0.60	7.59^c^ ± 0.02

Values are means and standard deviation of two determinations.

Value followed by the same superscript letters in a column are not significantly (*P* > 0.05) different.

Keys: A = Control (unfermented Moringa flour); B = 12 h fermented Moringa flour; C = 24 h fermented Moringa flour; D = 48 h fermented Moringa flour; E = 72 h fermented Moringa flour.

### Solubility of defatted Moringa seed flour at different temperatures

The solubility of fermented Moringa seed flour at different temperatures is shown in Table 4. The solubility at 60 and 80°C increased with increase in the fermentation time. Sample B (12 h fermentation) was found to be significantly higher in solubility at both 60 and 80°C. At 100°C, the solubility significantly decreased in value with increase in fermentation time except at 12 h fermentation time. The decrease could be due to denaturation of protein present in the flour as a result of high temperature (100°C). Fermentation time shows increase in the solubility of Moringa flour samples, especially at 60 and 80°C. Solubility of flour is an indication of its quality and digestibility therefore, Moringa flour would be suitable for incorporation to low soluble flour to improve solubility.

**Table 4:** Effect of fermentation time on the solubility of Moringa seed flour

Temperature (°C)	Solubility C				
	A	B	C	D	E
60	16.00d ± 0.00	107.00ª ± 7.07	73.50^b^ ± 2.12	41.50^c^ ± 2.12	66.00^b^ ± 2.83
80	45.00^c^ ± 1.41	74.50ª ± 2.12	65.00^b^ ± 1.14	67.00^b^ ± 4.24	67.00^b^ ± 1.41
100	91.00^b^ ± 1.41	105.00ª ± 4.24	68.00^c^ ± 0.00	67.00^c^ ± 1.41	70.00^c^ ± 2.83

Values are means and standard deviation of two determinations.

Value followed by the same superscript letters in a column are not significantly (*P* > 0.05) different.

Keys: A = Control (unfermented Moringa flour); B = 12 h fermented Moringa flour; C = 24 h fermented Moringa flour; D = 48 h fermented Moringa flour; E = 72 h fermented Moringa flour.

## Conclusion

Fermentation time showed beneficial effects on the functional and pasting properties of treated Moringa seed flour. Fermentation time significantly increased the water and oil absorption capacity as well as the emulsion capacity. However, dispersibility and bulk density were significantly reduced with fermentation time. These aspects imply the potential use as ingredient in food such as sauces, infant food, cakes, and bread products. The decreased in bulk density is desirable during flour packaging. Fermentation significantly increased trough viscosity, breakdown, final viscosity, setback, peak viscosity, and reduced the pasting time. The swelling power of the fermented flour showed significant increase at 60 and 100°C. The solubility of the flour was significantly affected at all temperatures, however, at 60°C, the samples had a significantly high solubility.

## Conflict of Interest

None declared.
